# Cocaine: What is the Crack? A Brief History of the Use of Cocaine as an Anesthetic

**DOI:** 10.5812/kowsar.22287523.1890

**Published:** 2011-09-26

**Authors:** Melody Redman

**Affiliations:** 1 Hull York Medical School, John Hughlings Jackson Building, University of York, Heslington, York, England

**Keywords:** Cocaine, Anesthesia, Anesthetics

Throughout the history of medicine, various drugs and technologies have been developed, and these developments have tremendously contributed to a range of treatments that are now taken for granted. It is only when we take time to study the development of these important drugs and technologies that we really realize how much effort and dedication went into developing them. Cocaine is an example of a drug that had many contributors aiding its use in medicine. Here, I discuss the factors and people that have contributed to the successful use of cocaine in anesthesia. Cocaine is an ancestor of modern-day anesthetics, although some perceive it as a drug associated with the dark and damaged segment of our society.

Cocaine is obtained from coca leaves. Although it was not isolated from coca leaves until the 19th century, its effects had made an impression in some parts of the world long before. The Incas knew the effects of chewing coca leaves many hundreds of years ago. In 1551, Peruvian bishops tried to persuade the government to prohibit coca use because chewing these leaves conflicted with their religious beliefs. Although the use of coca was not prohibited, the amount of land used for coca cultivation later in that decade was restricted; thus, the efforts of these bishops were not entirely in vain. It is interesting to note that similar to the history of chewing marijuana and tobacco, the history of chewing coca shows how a natural substance can be developed into a substance that is harmful and detrimental to both the individual and society. It was not until 1860 that Albert Niemann isolated cocaine from coca leaves. It has been reported that he “noticed that when he placed the crystals on his tongue, it made his tongue feel numb” (1). This could have been an early recognition of cocaine’s anesthetic properties. However, only in later years was this “numbness” recognized for its value in local anesthetics. Around 1868, Moreno y Maiz injected cocaine into a frog’s legs and conducted tests that lead him to propose the possibility of using cocaine as a local anesthetic in the future (1). Although this may seem like a significant breakthrough, in retrospect, it appears that Moreno y Maiz’s findings did not spark much interest in the public. Cocaine was not the first substance to be suggested as an anesthetic. Nitrous oxide, ether, and chloroform were recognized as anesthetics since 1844, 1846, and 1847, respectively. With these 3 substances being recognized for their anesthetic use a few years before the recognition of cocaine, it was quite some time before cocaine was first used for anesthesia because the most important developments in the anesthetic use of cocaine occurred in 1880s. Nowadays, such a delay would be expected because a drug has to undergo many trials and levels of testing before it can be licensed. However, at that time, such assessments were not regulated in the way they are today, and the use of cocaine as an anesthetic might have been delayed because of poor circulation of knowledge, lack of insight into its effects, or simply a lack of interest or facilities to explore it.

In 1880, Vassily von Anrep published a paper in which he suggested the use of cocaine for surgery (2). It has been suggested that von Anrep might have used cocaine for nerve block long before Halsted did (3). However, sufficient evidence for this suggestion is lacking, and therefore, Halsted is still reported as the first person to administer cocaine for nerve block. von Anrep appears to have not publicized his work enough; therefore, he did not get as much acknowledgment as he might have deserved, as so often is the case with medical discoveries. The year 1884 was significant in the history of cocaine’s use as an anesthetic in terms of its rise to fame. Sigmund Freud recognized his colleagues’ work on cocaine and published several reports on his own work on the drug. In Uber Coca, published in July 1884, Freud noted that “a first dose or even repeated doses of coca produce no compulsive desire to use the stimulant further; on the contrary, one feels a certain unmotivated aversion to the substance” (4). He also reported the effects of cocaine on the feelings and pulse rate of an individual and the duration of these effects. This study can be considered as a clinical trial in its most simple form, and although according to present knowledge, his results are erroneous, this paper provides a rather fascinating account of Freud’s beliefs about the substance. This paper also serves as a lesson from history regarding the importance of conducting precise trials in that a drug should be brought into widespread use on the basis of the knowledge of its various effects and not just on the basis of 1 person’s firsthand experience.

During that same year, Dr. Carl Koller investigated the anesthetic effects of cocaine on the eye. Koller’s work included the use of cocaine in cataract extraction and iridectomy (5). Whether the discoveries of Koller and Freud overlapped remains a matter of debate, but Koller presented his results at the Medical Society of Vienna on October 25, 1884 (6); this was a significant step contributing to the use of cocaine as an anesthetic.

In 1884, cocaine was used for the first time for nerve block by William Halsted. Halsted had a great influence on modern surgery, and notably, he was one of the founders of the Johns Hopkins School of Medicine. He made many remarkable discoveries in the field of anesthesia and was a pioneer in the field. However, along with several other colleagues, Halsted became addicted to cocaine later that year (7) after the famous mandibular nerve block. This is understandable when you consider that “The physicians and students experimented by performing nerve blocks on each other, only then administering the drug in a similar manner to patients” (7). Although Halsted’s discovery was remarkable, it had a detrimental effect on him, leading to a serious addiction; some believe this was followed by an addiction to morphine, which was initially used to wean him off cocaine (7). However, when one takes into account the fascination with the use of cocaine as an anesthetic, one cannot help but question as to whether Halsted was really aware of the addictive properties of cocaine before he became addicted to it.

In 1885, the next big step occurred. James Corning, a neurologist from America, had learned a lot about spinal anatomy in Europe (8). Some of Corning’s knowledge about the spine’s anatomy was incorrect (8), but he had enough understanding of the subject to perform the first spinal anesthesia, a revolutionary procedure of his time. The procedure successfully produced loss of both pain and sensation in one of Corning’s patients who had spinal weakness. The effects lasted for around 30 min in the patient, and the only side effects were headache and dizziness (8). In 1886, Corning published Local Anesthesia in general medicine and surgery, the first textbook of its kind. A lot of the information provided in this book was closely linked to Halsted’s work. Corning was Halsted’s student, but on reading his book containing statements such as “Thus in a number of experiments made by Dr. Halsted and myself, we have found that,” one may see them as colleagues (9). Corning might have appeared to be taking more credit than Halsted felt was due (7). Nonetheless, the importance of this textbook in publicizing the anesthetic use of cocaine is greatly recognized.

The technique of lumbar puncture was developed in 1891. This led to the successful use of cocaine as a spinal anesthetic by Dr. August Bier in 1898. The following year, he reported the method he adopted for performing 6 unproblematic orthopedic operations in his paper entitled “Experiments with cocainization of the spinal cord. ” In addition, he theorized the manner in which his method might have affected the nerves and spinal cord (8). Throughout the 1890s, doctors administered inappropriate doses of cocaine to patients causing toxicity, which was potentially fatal. Over the turn of the century, knowledge of the effects of cocaine increased, and unsurprisingly, the number of doctors who encouraged its use decreased, leading to a reduction in the reported number of cocaine-associated deaths.

As is the case with most medical discoveries, the use of cocaine became a part of the popular culture of that time. Various drinks (including wine) and other products had coca, with the perceived positive medical qualities of cocaine being an extra subject for advertisement. Perhaps, one of the most famous drinks with coca was Coca-Cola®, which was patented in 1887 (1). Until 1903, this drink contained cocaine. The increasing number of reports on the adverse effects of cocaine prompted the manufacturers of Coca-Cola to adopt a process for removing cocaine from the coca leaves while leaving the essential oils in the drink. This is just an example of how overflow of information from the field of medicine into society can be exploited by industries.

Between 1890 and 1910, a number of important advances were made in both technology, such as development of portable cameras, electronic typewriters, and automobiles, and medicine, such as the discovery of x-rays and radiation. As the developed world was blossoming with these technological advancements, advancements in local anesthetics were not focused upon. It is quite possible that all these developments were changing the world and contributed to the use of cocaine as a street drug by people for coping with these changes. Throughout history, addictions among people have been focused on some substance or phenomenon, be it alcohol or behaviors. In the early 1900s, cocaine addiction was rampant since consumption of cocaine was new and unexplored. At that time, cocaine could be easily obtained from a local chemist or a street salesperson. Cocaine abuse is prevalent even today, but the problem had reduced for a while. The reduction in cocaine abuse was probably attributable to media’s coverage of the problem, particularly in America. In 1900–1920, both the New York Times and Journal of the American Medical Association reported that cocaine addiction among black people was leading to serious crimes (10). The detrimental effects of cocaine on the society were being recognized. This led to decreased use of cocaine, and by the 1930s, cocaine was largely substituted by amphetamine (11).

Further progress in the field of local anesthesia occurred. In 1905, procaine, a synthetic agent that was much safer than cocaine, was synthesized by Dr. Alfred Einhorn. Further, Heinrich Braun combined procaine with adrenaline to increase its duration of action (12). Since procaine was far less addictive than cocaine, procaine became a much more popular anesthetic than cocaine. During the following years, several other improved compounds were developed, and the medical use of cocaine reduced further. However, cocaine was an early anesthetic, and its use paved the path for the development of other anesthetics. Currently, cocaine is known mainly as a drug of abuse and was classified as a class A drug by the Misuse of Drugs Act 1971 (13). Although cocaine is still being widely researched upon, a large proportion of this research is focused on its effects in addiction and abuse and not on its use as an anesthetic.

In conclusion, the discovery of the anesthetic use of cocaine was quite remarkable and crucial for the development of the current local anesthetics. Many people were involved in the development of cocaine as an anesthetic, but the credit might not have been entirely attributed as due, as is the case for many medical discoveries. Cocaine was being used in medicine largely during the end of the 19th century, but the addictive property of cocaine made it less of a wonder drug than it was initially thought to be. Other synthetic compounds that were safer than cocaine replaced cocaine as anesthetics. Currently, in the UK, cocaine is rarely used in medicine, but sadly, it is one of the most abused drugs. Nonetheless, its medical use in the 19th century can be described as a major breakthrough in the field of anesthesia.
